# Supplementary material for: Active bacteria driving N2O mitigation and dissimilatory nitrate reduction to ammonium in ammonia recovery bioreactors
Source: ISME J. 2025 Feb 6;19(1):wraf021. doi: 10.1093/ismejo/wraf021 (PMC11879220; doi:10.1093/ismejo/wraf021)
Supplement: SUPPLEMENTARY_DATA_(V31)_wraf021 [file supplementary_data_(v31)_wraf021.docx]

**SUPPLEMENTARY INFORMATION**

**Active bacteria driving N_2_O mitigation and dissimilatory nitrate reduction to ammonium in ammonia recovery bioreactors**

Hop V. Phan, ^1^ Shohei Yasuda,^2, 3^ Kohei Oba,^1^ Hiroki Tsukamoto,^1^ Tomoyuki Hori,^4^ Megumi Kuroiwa,^1^ Akihiko Terada^1,2^

^1.^ Department of Applied Physics and Chemical Engineering, Tokyo University of Agriculture and Technology, 2-24-16 Naka-Cho, Koganei, Tokyo, 184-8588, Japan

^2.^ Global Innovation Research Institute, Tokyo University of Agriculture and Technology, 3-8-1 Harumi-Cho, Fuchu, Tokyo, 185-8538, Japan

^3.^ Department of Civil Engineering, University of Galway, University Road, Galway, H91 TK33, Ireland

^4.^ Environmental Management Research Institute, National Institute of Advanced Industrial Science and Technology, 16-1 Onogawa, Tsukuba, Ibaraki, 305-8569, Japan

*Corresponding author: [akte@cc.tuat.ac.jp](mailto:akte@cc.tuat.ac.jp)

**Text S1: Merits of metagenomics and metatranscriptomics approaches**

A hybrid sequencing approach with high-quality Illumina SR and Nanopore LR recovered 98 non-redundant MAGs (dereplication at 99% ANI), meeting the GTDB database criteria described in **2.5**. Only one recovered MAG belongs to *Archaea* (genus *Methanomassiliicoccus*); 97 non-redundant MAGs belong to *Bacteria*. Of them, 53 MAGs were above the criteria, having completeness > 90% and contamination < 5%. Average completeness and contamination were 85.7% and 1.6%, respectively. Fourteen MAGs were recovered with fewer than 10 contigs, and three of these MAGs contained only one contig (**Table S2**).

The 16S rRNA gene amplicon is a common method for profiling bacterial communities. However, this method often overestimates some microbial groups and *vice versa* for others, which is noted in this study and previous works (Xu et al., 2021). For example, a higher fraction of *Bacillota* and a lower fraction of *Chloroflexota* in the 16S rRNA gene profiles than in metagenomic profiles were consistently observed. A low copy number of the 16S rRNA gene in *Chloroflexota* (1.4 ± 0.7) and a high copy number of the 16S rRNA gene in *Bacillota* (6.9 ± 2.9) (<https://rrndb.umms.med.umich.edu/>) possibly explain for this difference. On the other hand, consistent with this study, a high number of MAGs belonging to *Bacteroidota* were reconstructed from activated sludge (Singleton et al., 2021), while the 16S rRNA gene amplicon study often observed a much higher abundance of *Proteobacteria* at a global scale (Wu et al., 2019). Bacterial groups lack culture representative (named “microbial dark matter”) and were at low presence in the reference database, thus not well captured by the 16S rRNA gene amplicon approach. For example, members of *Riflebacteria*, *Candidatus* Hinthialibacterota , and UBA10199 were only obtained in MAG profiles, but not in 16S rRNA gene amplicon profiles.

The discrepancies between the functional potential in metagenomic data and the actual expression in metatranscriptomic data in this study indicate the importance of combining both metagenomic and metatranscriptomic for studying the functions of the microbial community under specific conditions.

**Text S2: The predominant and active bacterial members of the MAS system**

Overall response of bacterial community members to the change in DO concentration was somewhat taxonomically explainable (**Fig. 2**). The downregulation was observed for 24 MAGs out of 30 MAGs in *Bacteroidota*; only 2 MAGs were upregulated and 4 MAGs did not significantly respond to the increase in DO concentration. In contrast, 14 out of 19 MAGs assigned to *Gammaproteobacteria* were significantly upregulated, only one MAG was downregulated, and the other 4 MAGs were not statistically changed. All *Alphaproteobacteria* (7 MAGs) and *Gemmatimonadota* members (4 MAGs) were upregulated after the increase in a DO concentration.

Consistent with the community-wide observation, the most downregulated bacterial members under the high DO condition were unclassified MAGs of *Bdellovibrionales* (R2_bin.6_o) and *Cloacimonadaceae* (R1_bin.36_r). The abundances of these MAGs were low (log_2_TPM of 9.5 for *Bdellovibrionales* MAG) and *moderately* low (log_2_TPM of 12.1 for *Cloacimonadaceae* MAG). Their transcriptomic activities were moderately high under the microaerophilic condition (log_2_TPM of 13.5 [*Bdellovibrionales*] and 13.7 [*Cloacimonadaceae*]) and significantly decreased (log_2_FC of -4.1 [*Bdellovibrionales*] and log_2_FC of -3.9 [*Cloacimonadaceae*]) at a high DO condition. Unclassified MAGs of UBA1426 (*Bacillota*), UBA8515 (*Patescibacteria*), and *Desulfomicrobium* (*Desulfobacterota*) displayed the comparable transcriptomic trend (**Fig. 2**).

The most upregulated member at the high DO concentration was an unknown species of *Pararhodobacter* (R1_bin.49_o) in *Alphaproteobacteria*. This member was moderately abundant (log_2_TPM of 13.8) but showed low activity under the microaerophilic condition (log_2_TPM of 7.9). Its relative activity significantly increased (log_2_FC of 5.4) under the high DO condition. The 10 most upregulated members (log_2_FC of 3.9 to 4.9) under the high DO condition include one MAG in *Verrumicrobiota* (*Opitutaceae*), six MAGs in *Alphaproteobacteria* (JaaKGP01*, Bosea,* UBA1943*, Wagnerdoeblera,* FKL33 genera), one MAG in *Actinomycetota* (*Leucobacter* sp. 002336855), and two MAGs in *Gammaproteobacteria* (CAISIP01 and *Ottowia* sp001897615) (**Fig. 2**).

**Test S3: Additional microorganisms harboring genes of terminal oxidases**

Except for *Cloacibacterium* sp. 002422665 (R1_bin.104_o), *Bacteroidota* UBA6192 (R2_bin.12_r), and *Chloroflexota* UBA8950 (R1_bin.54_o), several bacterial members actively expressing terminal oxidase and nitrogen metabolism genes were detected. *C. normanense* (R1_bin.29_r) was the second active member with lower gene expression (mean SCM of 3.7 times lower and *ccoNO* of 3.5 times lower than those of *Cloacibacterium* sp. 002422665). *Bacteroidota* CAISCU01 was another bacterium with high expressions of high-affinity *ccoNO* and clade II *nosZ,* which were significantly downregulated under elevated DO concentration. *Paludibacter* (R2_bin.117_r) was the third active member under a microaerophilic condition showing moderate expression for high-affinity oxidase *cydAB* (4.5 times of SCM) and *nrfA* (2.7 times of SCM). In addition, this bacterium had a high expression of *qnorB* gene (8 times of SCM). Thus, this bacterium was capable of utilizing different electron acceptors, particularly NO, for conserving energy. Increasing DO concentration inhibited the activity of this MAG (**Figs. 5 and 6**).

*Thermomonas* (R1_bin.8_o) utilized high-affinity *ccoNO* genes (expression > 83 times of SCM) as the primary strategy of energy conservation under a microaerophilic condition. Additionally, this bacterium had gene expression of low-affinity oxidases *coxAB* (> 3 times of SCM) (**Fig. 6**). Capability of utilizing both high-affinity and low-affinity terminal oxidases explained the preferential growth of *Thermomonas* bacterium under microaerophilic and alternative redox conditions.

Increasing DO concentration induced the expression of low-affinity oxidases, exclusively for *Proteobacteria* that possess neither clade II *nosZ* nor DNRA genes (*nrfA* and *octR*). These bacteria also harbored high-affinity oxidases (mainly *ccoNO*) with decreasing expression and other truncated denitrifying genes with low expressions (**Figs. 5** and **6**).

**Fig. S1**: Profiles of aeration rate, DO, NH_4_^+^-N, NO_3_^-^-N, and off-gas N_2_O of the MAS system (R2) on days 268 and 269. The red vertical dash lines indicate the time points of doubling and reducing the aeration rates, respectively. The red arrows represent the sampling points for metagenomics (before doubling an aeration rate) and metatranscriptomics (before and after doubling an aeration rate). Aeration volume, DO, NH_4_^+^-N, and NO_3_^-^-N were online recorded while off-gas N_2_O was manually sampled and measured by GC-MS.

**Fig. S2:** Unrooted phylogenetic tree of NosZ proteins. The tips are labeled with the taxonomy at the genus level. The color strip indicates the references of NosZ types, clade II NosZ (orange) and clade I NosZ (green). NosZ sequences in this study are left blank. Bootstrap values (>50) based on 1000 replications are shown at branch nodes. Reference sequences were selected from a previous study (Sanford et al., 2012) and downloaded from NCBI database (**Table S1**). Sequences were aligned using MUSCLE followed by tree prediction in IQTREE2. The best-fit model, according to BIC, was Q.pfam+R5. The tree scale represents 1.

**Fig. S3:** Phylogenetic tree of NrfA, OctR, and NirB proteins (unrooted). The tips are labeled with the taxonomy at the genus level. The color strip indicates the references of NirB (green), NrfA (orange), and OctR (purple). Sequences in this study are left blank. Bootstrap values (>50) based on 1000 replications are shown at branch nodes. Reference sequences for NrfA were selected from a previous study (Welsh et al., 2014). References for NirB and OctR were top Blastp hits downloaded from NCBI. NirB sequence from *E.coli* and OctR sequence from Shewanella are included. Accession numbers of all reference sequences are provided in **Table S1**. Sequences were aligned using MUSCLE followed by tree prediction in IQTREE2. The best-fit model, according to BIC, was **WAG+R5**. The tree scale represents 1.

**Fig. S4**: The taxonomy and genotypes of the genus *Cloacibacterium* (*Bacteroidota*). (A) Phylogenomic tree of all available genomes (completeness > 95% and contamination < 2%) and two MAGs recovered in this study (Orange color). *Weeksella massiliensis* was included as an outgroup. The tips were labeled with taxonomic affiliation classified by GTDB-tk (v2.4.0) and NCBI Genbank assembly accession numbers (in parenthesis). The tree scale represents 0.1. (B) The heatmap shows metabolic pathway completeness calculated by the KEGG Decoder. Each genome was labeled with NCBI GenBank assembly accession number.

**Fig. S5**: The taxonomy and genotypes of the genus UBA8950 (*Chloroflexota*). (A) Phylogenetic tree of all available genomes and one MAG recovered in this study (Orange color). The tips were labelled with taxonomic affiliation classified by GTDB-tk (v2.4.0) and NCBI Genbank assembly accession numbers in parenthesis. The tree scale represents 0.01. (B) The heatmap shows metabolic pathway completeness calculated using the KEGG Decoder. Each genome was labelled with NCBI GenBank assembly accession number.

**Fig. S6**: The taxonomy and genotypes of the genus UBA6192 (*Bacteroidota*). (A) Phylogenetic tree of all available genomes and one MAG recovered in this study (Orange color). The tips were labelled with taxonomic affiliation classified by GTDB-tk (v2.4.0) and NCBI Genbank assembly accession numbers in parenthesis. The tree scale represents 0.01. (B) The heatmap shows metabolic pathway completeness calculated using the KEGG Decoder. Each genome was labelled with NCBI GenBank assembly accession number.

**Fig. S7:** The taxonomy and genotypes of the genus UTCHB3 (*Bacteroidota*). (A) Phylogenomic tree of all available genomes and four MAGs recovered in this study (Orange color). The tips were labelled with taxonomic affiliation classified by GTDB-tk (v2.4.0) and NCBI Genbank assembly accession numbers in parenthesis. The tree scale represents 0.01. (B) The heatmap shows metabolic pathway completeness calculated using the KEGG Decoder. Each genome was labelled with NCBI GenBank assembly accession number.

**Table S1:** NCBI accession numbers of protein sequences employed to build the phylogenetic trees.

| NosZ references | |  | DNRA references | |
| --- | --- | --- | --- | --- |
| NCBI accession numbers | NosZ types |  | NCBI accession numbers | DNRA Types |
| WP_012632836 | Clade II NosZ |  | WP_001365050 | NrfA |
| WP_012140537 | Clade II NosZ |  | WP_011864650 | NrfA |
| WP_012844504 | Clade II NosZ |  | WP_011138866 | NrfA |
| WP_014792165 | Clade II NosZ |  | AUR45802 | NrfA |
| WP_004919511 | Clade II NosZ |  | WP_004514005 | NrfA |
| WP_011709298 | Clade II NosZ |  | ADH98815 | NrfA |
| WP_012962813 | Clade II NosZ |  | WP_010937928 | NrfA |
| CAM74903 | Clade II NosZ |  | WP_012634152 | NrfA |
| WP_012374624 | Clade II NosZ |  | CAG9061977 | NrfA |
| WP_014288022 | Clade II NosZ |  | WP_012797970 | NrfA |
| WP_011372928 | Clade II NosZ |  | AAN57117 | OctR |
| WP_011222995 | Clade I NosZ |  | WP_002931494 | OctR |
| WP_013879286 | Clade I NosZ |  | WP_015773582 | OctR |
| WP_012251172 | Clade I NosZ |  | MBN8545165 | OctR |
| WP_011083147 | Clade I NosZ |  | WP_000049227 | NirB |
| WP_011205031 | Clade I NosZ |  | WP_166156952 | NirB |
| WP_011750448 | Clade I NosZ |  | WP_121240131 | NirB |
| WP_011914573 | Clade I NosZ |  | WP_043741483 | NirB |
| WP_012430154 | Clade I NosZ |  |  |  |

**Table S2:** List of the retrieved MAGs. The version with a higher resolution can be acquired as an excel file.

**References**

SANFORD, R. A., WAGNER, D. D., WU, Q., CHEE-SANFORD, J. C., THOMAS, S. H., CRUZ-GARCÍA, C., RODRÍGUEZ, G., MASSOL-DEYÁ, A., KRISHNANI, K. K., RITALAHTI, K. M., NISSEN, S., KONSTANTINIDIS, K. T. & LÖFFLER, F. E. 2012. Unexpected nondenitrifier nitrous oxide reductase gene diversity and abundance in soils. *Proceedings of the National Academy of Sciences,* 109**,** 19709-19714.

WELSH, A., CHEE-SANFORD, J. C., CONNOR, L. M., LÖFFLER, F. E. & SANFORD, R. A. 2014. Refined NrfA Phylogeny Improves PCR-Based nrfA Gene Detection. *Applied and Environmental Microbiology,* 80**,** 2110-2119.
